# Supplementary material for: X-chromosome target specificity diverged between dosage compensation mechanisms of two closely related Caenorhabditis species
Source: eLife. 2023 Mar 23;12:e85413. doi: 10.7554/eLife.85413 (PMC10076027; doi:10.7554/eLife.85413)
Supplement: Figure 1—figure supplement 4—source data 1. [file elife-85413-fig1-figsupp4-data1.zip › Figure 1ΓÇöfigure supplement 4 source data/Figure 1ΓÇöfigure supplement 4 source data labeled.pdf]

Figure 1—figure supplement 4—source data labeled

A

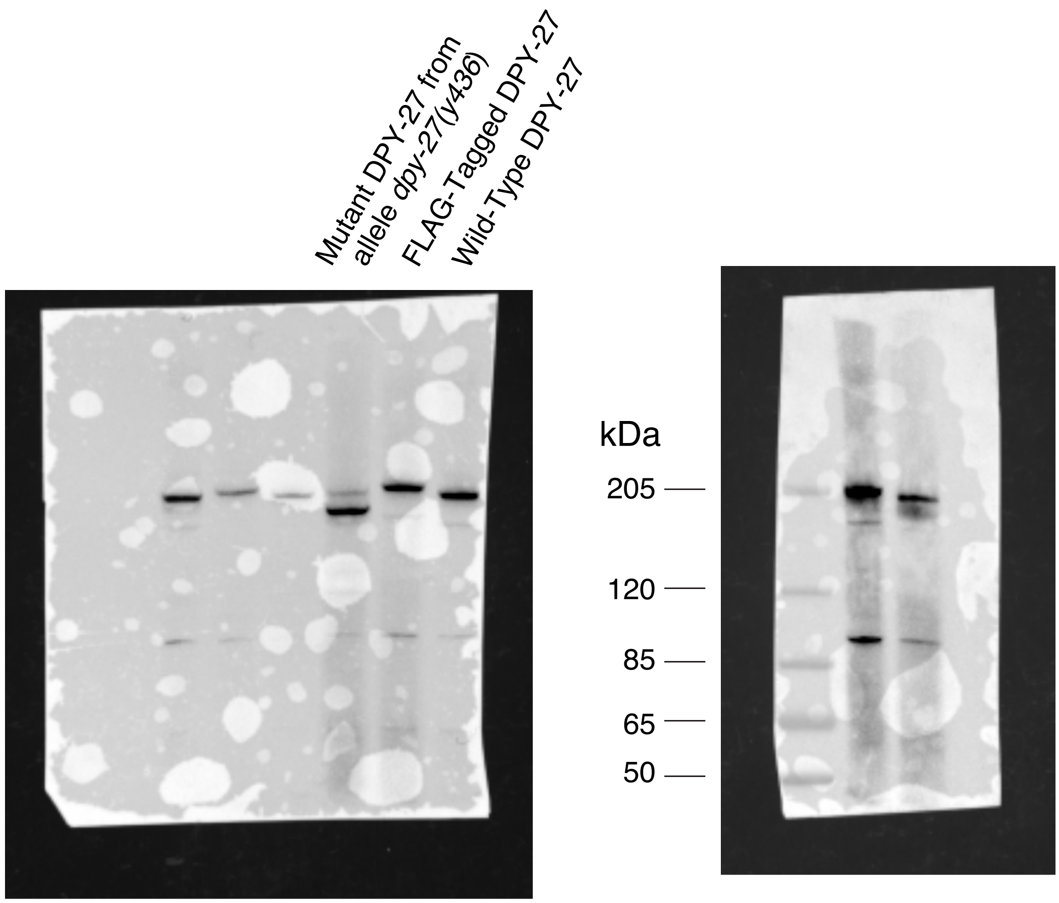

Merged images of chemilumenscence and visible channels
(These two blots derived from a single nitrocellulose membrane after the gel transfer.)

B

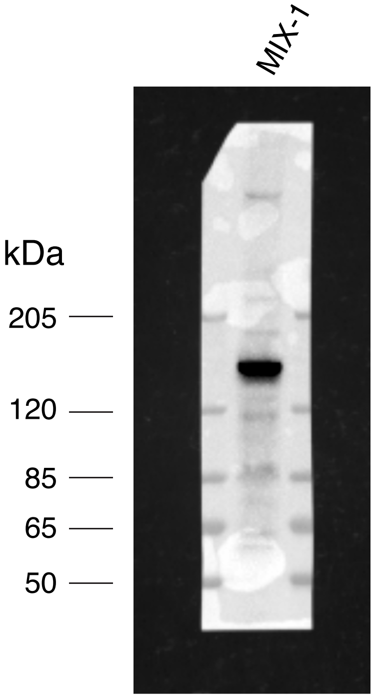

Merged image of chemilumenscence and visible channels
