# Supplementary material for: X-chromosome target specificity diverged between dosage compensation mechanisms of two closely related Caenorhabditis species
Source: eLife. 2023 Mar 23;12:e85413. doi: 10.7554/eLife.85413 (PMC10076027; doi:10.7554/eLife.85413)
Supplement: Supplementary file 1. [file elife-85413-supp1.docx]

| **Strain** | **Species** | **Genotype** | **Description** |
| --- | --- | --- | --- |
| TY5004 | *Cbr* | *mix-1(y435)* / + II | 460 bp deletion (Δ 5’ UTR into 2^nd^ intron, out of frame), null allele |
| TY5005 | *Cbr* | *dpy-27(y436)* / + III | 632 bp deletion (Δ 5' UTR, exon 1, intron 1, and part of exon 2), null |
| TY5006 | *Cbr* | *xol-1(y430)* X | 589 bp deletion (Δ promoter into 2^nd^ exon, out of frame), null |
| TY5153 | *Cbr* | *dpy-27(y436)* / + III; *xol-1(y430)* X |  |
| TY5230 | *Cbr* | *xol-1(y430) sdc-2(y453)* / + *xol-1(y430)* X | *sdc-2* null deletion allele created by genome editing |
| TY5231 | *Cbr* | *xol-1(y430) sdc-2(y454)* / + *xol-1(y430)* X | *sdc-2* null deletion allele created by genome editing |
| TY5232 | *Cbr* | *xol-1(y430) sdc-2(y455)* / + *xol-1(y430)* X | *sdc-2* null deletion allele created by genome editing |
| TY5237 | *Cbr* | *xol-1(y430) sdc-2(y460)* / + *xol-1(y430)* X | *sdc-2* null deletion allele created by genome editing |
| TY5363 | *Cbr* | *sdc-2(y467)* / + X | *sdc-2* null deletion allele created by genome editing |
| TY5365 | *Cbr* | *sdc-2(y469)* / + X | *sdc-2* null deletion allele created by genome editing |
| TY5753 | *Cel* | *dpy-27(y679)* III | 3xFlag-tagged *Cel dpy-27* |
| TY5773 | *Cbr* | *dpy-27(y705)* / + III | 52 bp deletion in exon 4 (Δ starts at codon 689, out of frame), null |
| TY5774 | *Cbr* | *dpy-27(y706)* III | 3xFlag-tagged *Cbr dpy-27* |
| TY5775 | *Cbr* | *sdc-2(y716)* X | 3xFlag-tagged *Cbr sdc-2* |
| TY5836 | *Cbr* | *dpy-27(y706)* III; *rex-1(y747)* X | *Cbr* MEX II (-27.58) scrambled at *Cbr rex-1* |
| TY5837 | *Cbr* | *dpy-27(706)* III; *rex-1(y749)* X | 5 motifs (4 *Cbr* MEX and 1 *Cbr* MEX II) scrambled in *Cbr rex-1* |
| TY5847 | *Cel* | *dpy-27(y679)* III; *yIs185 (Cbr rex-1)* X | *Cbr rex-1* insertion at *Cel* site 2 |
| TY5852 | *Cel* | *dpy-27(y679)* III; *yIs176 (Cbr rex-2)* X | *Cbr rex-2* insertion at *Cel* site 2 |
| TY5854 | *Cel* | *dpy-27(y679)* III; *yIs187 (Cel rex-32)* X | *Cel rex-32* insertion at *Cel* site 2 |
| TY5862 | *Cel* | *dpy-27(y679)* III; *yIs193 (Cbr rex-9)* X | *Cbr rex-9* insertion at *Cel* site 2 |
| TY5863 | *Cel* | *dpy-27(y679)* III; *yIs194 (Cbr rex-7)* X | *Cbr rex-7* insertion at *Cel* site 2 |
| TY5865 | *Cel* | *dpy-27(y679)* III; *yIs195 (Cbr rex-5)* X | *Cbr rex-5* insertion at *Cel* site 2 |
| TY5942 | *Cel* | *dpy-27(y679)* III; *yIs204 (Cbr rex-4)* X | *Cbr rex-4* insertion at *Cel* site 2 |
| TY5945 | *Cbr* | *sdc-2(y716)* *rex-4(y799)* X | *Cbr rex-4* MEX (-13.8) scrambled |
| TY5975 | *Cbr* | *sdc-2(y716)* *rex-4(y824)* X | *Cbr rex-4* MEX (-13.8) scrambled and MEX II (-19.09) scrambled |
| TY5976 | *Cbr* | *sdc-2(y716)* r*ex-4(y825)* X | *Cbr rex-4* MEX II (-19.09) scrambled |
| TY6075 | *Cbr* | *sdc-2(y716)* *rex-3(y849)* X | *Cbr rex-3* MEX II (-12.36) scrambled |
| TY6076 | *Cbr* | *sdc-2(y716) rex-3(y850)* X | *Cbr rex-3* MEX II (-20.04) scrambled |
| TY6121 | *Cbr* | *sdc-2(y716) rex-3(y868)* X | *Cbr rex-3* MEX II (-12.36) scrambled and MEX II (-20.04) scrambled |
| TY5946 | *Cbr* | *sdc-2(y716) rex-7(y800)* X | *Cbr rex-7* MEX (-18.72) scrambled |
| TY6072 | *Cbr* | *sdc-2(y716)* *rex-7(y846)* X | *Cbr rex-7* MEX (-18.72) scrambled and MEX (-12.26) scrambled |
| TY6085 | *Cbr* | *sdc-2(y716) rex-7(y855)* X | *Cbr rex-7* MEX (-18.72) scrambled and MEX (-12.58) scrambled |
| TY6086 | *Cbr* | *sdc-2(y716)* *rex-7(y857­­)* X | *Cbr rex-7* MEX (-18.72) scrambled, MEX (-12.26) scrambled,  and MEX (-12.58) scrambled |
| TY4573 | *Cel* | *sdc-2(y74)* X; *yEx992* | Expression of *3xflag::sdc-2* from an extrachromosomal array for the *in vitro* assay |
| TY6122 | *Cel* | *rex-33(y869)* X | 3 *Cel* MEX motifs replaced by *Cbr* MEX motifs in *Cel rex-33* |
| TY6123 | *Cel* | *rex-33(y870)* X | 3 *Cel* MEX motifs scrambled |
| TY6142 | *Cel* | *rex-33(y874)* X | 3 *Cel* MEX motifs replaced by *Cbr* MEX with G7C substitution in *Cel rex-33* |
| TY6143 | *Cel* | *rex-33(y875)* X | 3 *Cel* MEX motifs with C4G substitution |
| TY6106 | *Cel* | *rex-39(y861)* X | 2 *Cel* MEX II motifs replaced by *Cbr* MEX II motifs in *Cel rex-39* |
| TY5759 | *Cel* | *rex-39(y686)* X | 2 *Cel* MEX II motifs scrambled |
| AF16 | *Cbr* | wild-type *C. briggsae* |  |
| JU935 | *Cbr* | *mfIs27(Ce-lip-1::gfp, Ce-myo-2::gfp)* X | Used to determine parental origin of *Cbr* X chromosome |

All strains in this table except TY4573, AF16, and JU935 were created in this study.
