# Supplementary material for: X-chromosome target specificity diverged between dosage compensation mechanisms of two closely related Caenorhabditis species
Source: eLife. 2023 Mar 23;12:e85413. doi: 10.7554/eLife.85413 (PMC10076027; doi:10.7554/eLife.85413)
Supplement: Supplementary file 2. [file elife-85413-supp2.docx]

| **Target** | **Figure** | **Location** | **Primer name** | **Sequence** | **Function** |
| --- | --- | --- | --- | --- | --- |
| *Cbr rex-3* | Figure 10, Figure 10—Figure supplement 1 | A | QY171 | GATGATGAACTAAATCGTAAGCTTCC | qPCR for DCC binding |
|  |  | B | QY172r | CAGGGAAGATTAACTTGAAACTTCAG | qPCR for DCC binding |
| *Cbr rex-3* | Figure 10, Figure 10—Figure supplement 1 | C | QY173 | GCCTCAGGTCTTACGGTAGAAG | qPCR for DCC binding |
|  |  | D | QY174r | CTCAGAGACTTTTTGTACATTGTATTTG | qPCR for DCC binding |
| *Cbr rex-3* | Figure 10, Figure 10—Figure supplement 1 | D | QY175 | CAAATACAATGTACAAAAAGTCTCTGAG | qPCR for DCC binding |
|  |  | E | QY176r | CTAGCTTGCACATCAAGAAGAC | qPCR for DCC binding |
| *Cbr rex-3* | Figure 10, Figure 10—Figure supplement 1 | E | QY157 | GTCTTCTTGATGTGCAAGCTAG | qPCR for DCC binding |
|  |  | F | QY177r | CACGTTTCTATTAAACATTTCCTC | qPCR for DCC binding |
| *Cbr rex-3* | Figure 10, Figure 10—Figure supplement 1 | F | QY010 | GAGGAAATGTTTAATAGAAACGTG | qPCR for DCC binding |
|  |  | G | QY011r | CTTTGCATATGTCCTTTCACG | qPCR for DCC binding |
| *Cbr rex-3* | Figure 10, Figure 10—Figure supplement 1 | G | QY178 | CGTGAAAGGACATATGCAAAG | qPCR for DCC binding |
|  |  | H | QY156r | GCTATTCGACAAACACTCCACAC | qPCR for DCC binding |
| *Cbr rex-3* | Figure 10, Figure 10—Figure supplement 1 | H | QY179 | GTGTGGAGTGTTTGTCGAATAGC | qPCR for DCC binding |
|  |  | I | QY180r | CCCAATATGTTCCGTTTCTTACTG | qPCR for DCC binding |
| *Cbr rex-3* | Figure 10, Figure 10—Figure supplement 1 | J | QY181 | GGTTACCTAACGGAAATCCTGTG | qPCR for DCC binding |
|  |  | K | QY182r | GATCGTAAATGCACACATGCATTC | qPCR for DCC binding |
| *Cbr rex-4* | Figure 9, Figure 9— Figure supplement 1 | A | QY121 | GGACTGTGCTCTGGCG | qPCR for DCC binding |
|  |  | B | QY122r | GGGACCATGGTTACTTTTCTTG | qPCR for DCC binding |
| *Cbr rex-4* | Figure 9, Figure 9— Figure supplement 1 | C | QY123 | GAAAAGTTAACGCTCCGCTC | qPCR for DCC binding |
|  |  | D | QY124r | GTCAGTTGACCTTACTCATTCAG | qPCR for DCC binding |
| *Cbr rex-4* | Figure 9, Figure 9— Figure supplement 1 | D | QY125 | CTGAATGAGTAAGGTCAACTGAC | qPCR for DCC binding |
|  |  | E | QY126r | GAATGCCATACGATGTCTGAC | qPCR for DCC binding |
| *Cbr rex-4* | Figure 9, Figure 9— Figure supplement 1 | E | QY012 | GTCAGACATCGTATGGCATTC | qPCR for DCC binding |
|  |  | F | QY013r | GTAGCAGGCCACTAGTTTCC | qPCR for DCC binding |
| *Cbr rex-4* | Figure 9, Figure 9— Figure supplement 1 | F | QY127 | GGAAACTAGTGGCCTGCTAC | qPCR for DCC binding |
|  |  | G | QY128r | CTATCTTCGCAGAAAGTCTGAC | qPCR for DCC binding |

| **Target** | **Figure** | **Location** | **Primer name** | **Sequence** | **Function** |
| --- | --- | --- | --- | --- | --- |
| *Cbr rex-4* | Figure 9, Figure 9— Figure supplement 1 | G | QY129 | GTCAGACTTTCTGCGAAGATAG | qPCR for DCC binding |
|  |  | H | QY130r | CCATAGAACATAGTTCCTGGTTC | qPCR for DCC binding |
| *Cbr rex-4* | Figure 9, Figure 9— Figure supplement 1 | H | QY131 | GAACCAGGAACTATGTTCTATGG | qPCR for DCC binding |
|  |  | I | QY132r | GAAAACATTGCGAAGACTCAAC | qPCR for DCC binding |
| *Cbr rex-4* | Figure 9, Figure 9— Figure supplement 1 | J | QY133 | CTTTGGAAAGTCAGTTCCTC | qPCR for DCC binding |
|  |  | K | QY134r | CATGAATAGTATGTGCAGTGATG | qPCR for DCC binding |
| *Cbr rex-7* | Figure 11, Figure 11—Figure supplement 1 | A | QY135 | GATGTTGCTCTATTCAAAATGCG | qPCR for DCC binding |
|  |  | B | QY136r | CATAGATGCGGGATTTTTTGTG | qPCR for DCC binding |
| *Cbr rex-7* | Figure 11, Figure 11—Figure supplement 1 | C | QY018 | CATTGCAATAAACTGGTGGG | qPCR for DCC binding |
|  |  | D | QY019r | GCAGGGGATTAAGACAACATT | qPCR for DCC binding (MEX -12.26 wt) |
|  |  | D | QY190r | ACGCGAGATTAAGACAACATT | qPCR for DCC binding (MEX -12.26 scr) |
| *Cbr rex-7* | Figure 11, Figure 11—Figure supplement 1 | D | QY137 | AATGTTGTCTTAATCCCCTGC | qPCR for DCC binding (MEX -12.26 wt) |
|  |  | D | QY191 | AATGTTGTCTTAATCTCGCGT | qPCR for DCC binding (MEX -12.26 scr) |
|  |  | E | QY138r | GACTTGTAGAATCCTTTTTATCGC | qPCR for DCC binding |
| *Cbr rex-7* | Figure 11, Figure 11—Figure supplement 1 | E | QY139 | GCGATAAAAAGGATTCTACAAGTC | qPCR for DCC binding |
|  |  | F | QY140r | TAACACGTCTCCTATCACTC | qPCR for DCC binding |
| *Cbr rex-7* | Figure 11, Figure 11—Figure supplement 1 | G | QY141 | GGTTTTATGGCCGTGGTG | qPCR for DCC binding |
|  |  | H | QY142r | GCTATTCGAACGTCGAACAG | qPCR for DCC binding |
| *Cbr rex-1* | Figure 9, Figure 9— Figure supplement 1, Figure 10, Figure 10—Figure supplement 1, Figure 11, Figure 11—Figure supplement 1 |  | QY006 | CCCTTCCACTCTAGTCTAATCG | qPCR for DCC binding normalization |
|  |  |  | QY007r | GGTGTGTTTGATGATGTAGGC | qPCR for DCC binding normalization |
| *Cbr rex-2* | Figure 9, Figure 9— Figure supplement 1, Figure 10, Figure 10—Figure supplement 1, Figure 11, Figure 11—Figure supplement 1 |  | QY040 | CAAATTTGATCGAGTCAACCTC | qPCR for DCC binding normalization |
|  |  |  | QY041r | GAAAAGGAGAGTTATCACTCAATG | qPCR for DCC binding normalization |
| **Target** | **Figure** | **Location** | **Primer name** | **Sequence** | **Function** |
| *Cbr rex-5* | Figure 9, Figure 9— Figure supplement 1, Figure 10, Figure 10—Figure supplement 1, Figure 11, Figure 11—Figure supplement 1 |  | QY014 | CGAAGAAAGCATATGAAAGC | qPCR for DCC binding normalization |
|  |  |  | QY015r | CTCTAAAATAATTGTCCTCCGTC | qPCR for DCC binding normalization |
| *Cbr rex-9* | Figure 9, Figure 9— Figure supplement 1, Figure 10, Figure 10—Figure supplement 1, Figure 11, Figure 11—Figure supplement 1 |  | QY022 | GATACGAACAGGGTGCAAGG | qPCR for DCC binding normalization |
|  |  |  | QY023r | TCACATACTCGTTTCGTCCG | qPCR for DCC binding normalization |
| *Cbr* X negative control | Figure 9, Figure 9— Figure supplement 1, Figure 10, Figure 10—Figure supplement 1, Figure 11, Figure 11—Figure supplement 1 |  | QY099 | CCAGAAATAGCTATTCTAAGAGG | qPCR for negative control |
|  |  |  | QY100r | GTTTTTGAGTTCCCTGGCAC | qPCR for negative control |
| *Cel rex-8* | Figure 5, Figure 12, Figure 13 |  | rex8-F | TTTATCCACCAACATGCATAAG | qPCR for DCC binding normalization |
|  |  |  | rex8-R | CAGTGGATAACTACACAAGGG | qPCR for DCC binding normalization |
| *Cel rex-14* | Figure 12, Figure 13 |  | rex14-F | ACCTCCTTTCACAACACTCTTT | qPCR for DCC binding normalization |
|  |  |  | rex14-R | TCGAACCCAACTCGTTTATCTC | qPCR for DCC binding normalization |
| *Cel rex-16* | Figure 5, Figure 12, Figure 13 |  | rex16-F | GTACAAACGCAGGGAAGAGA | qPCR for DCC binding normalization |
|  |  |  | rex16-R | GACGCTACCACACCTTCAATA | qPCR for DCC binding normalization |
| *Cel rex-32* | Figure 5, Figure 12, Figure 13 |  | rex32-F | CACTCCCCAGCTAATTTGGA | qPCR for DCC binding normalization |
|  |  |  | rex32-R | TTCCCTTGTTGCGGAGATAG | qPCR for DCC binding normalization |
| *Cel rex-33* | Figure 12, Figure 13 |  | QY212 | GTGTGTTGCTGCCAAAGCCTG | *Cel rex-33* mutagenesis genotyping, sequencing |
|  |  |  | QY243 | GCAAGCACAGACACTCAAAC | qPCR for DCC binding |
|  |  |  | QY213r | GGGCCCGTGGTTAATTTATTCG | *Cel rex-33* mutagenesis genotyping; sequencing; qPCR for DCC binding |
| *Cel rex-35* | Figure 5, Figure 12, Figure 13 |  | rex35-F | CCATATGTTGCCCAATGTTCC | qPCR for DCC binding normalization |
|  |  |  | rex35-R | CGCAGGGAACATCAAATTAGTC | qPCR for DCC binding normalization |
| *Cel rex-36* | Figure 12, Figure 13 |  | rex36-F | CCCTCTTCAGGCGATAAATG | qPCR for DCC binding normalization |
|  |  |  | rex36-R | CGTTCATGCGAATGTCTCTC | qPCR for DCC binding normalization |
| *Cel rex-39* | Figure 12 |  | QY210 | CGATACATTTGTTTTTTATTAAATATCTACATTTCTCG | *Cel rex-39* mutagenesis genotyping; sequencing |
|  |  |  | QY211r | TTTCTGAAAAAATTGAAAGAATCTTGCTTAAAATG | *Cel rex-39* mutagenesis genotyping; sequencing |
|  |  |  | QY215 | AATGCACTCATGCACATGTTTC | qPCR for DCC binding |
|  |  |  | QY216r | CACAACAAGACCGAATAAATATAACAC | qPCR for DCC binding |
| *Cel rex-48* | Figure 5, Figure 12, Figure 13 |  | rex-48-F | CTGCGCGATAGGCAATAGT | qPCR for DCC binding normalization |
|  |  |  | rex-48-R | GCACAATTCCAAGTCATCCATAC | qPCR for DCC binding normalization |
| *Cel* site 2 | Figure 5 |  | ER589 | CAGCGTAGTTGCTGACACTTAATGGTTC | qPCR for DCC binding normalization |
|  |  |  | ER590 | CTTTTAAGCAGTCGTCATGTACGTGTTCG | qPCR for DCC binding normalization |
| *Cel* Chr I control | Figure 5, Figure 12, Figure 13 |  | autosome-F | ACCCACGACATTGCTCTTGT | qPCR for DCC binding normalization |
|  |  |  | autosome-R | AGTTTTGGGGCAGCTCTCTC | qPCR for DCC binding normalization |
| *Cel* X site 2 | Figure 5 |  | ER573 | CGTGCCAGTTGTTGACTTATG | *Cel* X site 2 insertion genotyping; sequencing |
|  |  |  | ER574 | CATGTTTTTGGCGCTGGTGAGTAGG | *Cel* X site 2 insertion genotyping; sequencing |
| *Cbr ben-1* |  |  | BF-2041 | GCTCGCTTTCTTTCCAAAAACGAGCAGAAGCCCCAATCGGTCG | Cas9 co-conversion marker for *Cbr* |
|  |  |  | BF-2042 | CGTGCGCAGCTTGTGATTCATGCTCCGCCCACTTTTCCG | Cas9 co-conversion marker for *Cbr* |
| *Cbr  dpy-27(y436)* | Figure 1F |  | CBDPY27.OL | GACGACAGAGTGGCTCTGCCGACAAGAGC | *Cbr* deletion library screening |
|  |  |  | CBDPY27.IL | GCCAACTTGCCGAATTTGAGC | *Cbr* deletion library screening |
|  |  |  | CBDPY27.PL | GGAGCTGTTGGAAGACTCGAGTGGTTGG | *Cbr* deletion library screening |
|  |  |  | CBDPY27.OR | CTTACAATGTCTTCAATCTGTTGGAAAAG | *Cbr* deletion library screening |
|  |  |  | CBDPY27.IR | GGCCATTTTGATCGTCGTTGTGG | *Cbr* deletion library screening |
|  |  |  | CBDPY27.PR | CCAGACGTCAATCTCAGCGATGAC | *Cbr* deletion library screening |
| *Cbr xol-1* | Figure 2 |  | CBXOL1.OL | GCCTAGTTTCACGTATTTCTCTAC | *Cbr* deletion library screening |
|  |  |  | CBXOL1.IL | GTAAGGCCAACCGGATTAGC | *Cbr* deletion library screening |
|  |  |  | CBXOL1.PL | CGCTTCAAGGAGACGCCGAGC | *Cbr* deletion library screening |
|  |  |  | CBXOL1.OR | CCCCGTGAAAAGAGTCTGCC | *Cbr* deletion library screening |
|  |  |  | CBXOL1.IR | CGGCACTTCTGGGTTTAGACG | *Cbr* deletion library screening |
|  |  |  | CBXOL1.PR | CGCATGTTCCTATGCAAACTTTGGC | *Cbr* deletion library screening |
| *Cel dpy-10* |  |  | BF-1853 | CGAACGTTCTCGCTGACAACGAACTATTCGCGTCAG | Cas9 co-conversion marker for *Cel* |
|  |  |  | BF-1854 | GCATGTTTGATTTGGAGTAGTTCCTGGCATTCC | Cas9 co-conversion marker for *Cel* |
