# Supplementary material for: X-chromosome target specificity diverged between dosage compensation mechanisms of two closely related Caenorhabditis species
Source: eLife. 2023 Mar 23;12:e85413. doi: 10.7554/eLife.85413 (PMC10076027; doi:10.7554/eLife.85413)
Supplement: Supplementary file 3. [file elife-85413-supp3.docx]

**Supplementary File 3. Chromosome-specific BACs used to generate FISH probes**

| **BAC** | **Chromosome** | **Start** | **End** |
| --- | --- | --- | --- |
| RPCI94_19F11 | III | 35266 | 135375 |
| RPCI94_21C16 | III | 53199 | 124610 |
| RPCI94_27L20 | III | 241669 | 341069 |
| RPCI94_27P10 | X | 4191 | 96616 |
| RPCI94_03E18 | X | 217136 | 321081 |
| RPCI94_01B13 | X | 237728 | 343819 |
| RPCI94_20J22 | X | 720980 | 839475 |
| RPCI94_28F15 | X | 1198052 | 1322029 |
| RPCI94_19L23 | X | 3179427 | 3291372 |
| RPCI94_19O24 | X | 14257820 | 14399879 |
| RPCI94_26I06 | X | 15980691 | 16042072 |
| RPCI94_28L18 | X | 16743266 | 16871287 |
| RPCI94_22H01 | X | 19865754 | 19995983 |
