# Supplementary material for: X-chromosome target specificity diverged between dosage compensation mechanisms of two closely related Caenorhabditis species
Source: eLife. 2023 Mar 23;12:e85413. doi: 10.7554/eLife.85413 (PMC10076027; doi:10.7554/eLife.85413)
Supplement: Supplementary file 4. [file elife-85413-supp4.docx]

**Supplementary File 4. List of target-specific sequences for guide RNAs used in CRISPR / Cas9 genome editing experiments**

| **Target** | **Figure** | **Target sequence (5’ to 3’)** | **Coordinates** | **Guide name** |
| --- | --- | --- | --- | --- |
| *Cbr dpy-27(y705)* | Figure 1F | CGCTCTGGAGTACGGTAAAA | III: 2729405..2729386 | *cbr-dpy-27* |
| *Cbr ben-1* | Figure 1, Figure 9, Figure 10, Figure 11 | CAACCTGATGGAACCTACAA | III: 8377724..8377705 | crispr_bf39 |
| *Cbr rex-4* | Figure 9, Figure 9—Figure supplement 1 | GCGCGTATGGCCAATTGGCA | X: 6358568..6358587 | crispr_bf80 |
| *Cbr rex-4* | Figure 9, Figure 9—Figure supplement 1 | TTTTATATGAACAGGGTGCG | X: 6358623..6358642 | crispr_bf77 |
| *Cbr rex-7* | Figure 9, Figure 9—Figure supplement 1 | TCTGAGATTTTATATGGGCA | X: 8026313..8026332 | crispr_bf79 |
| *Cbr rex-7* | Figure 11, Figure 11—Figure supplement 1 | CGAAGAGAAGAATGCGGCAG | X: 8026442..8026423 | crQY011 |
| *Cbr rex-7* | Figure 11, Figure 11—Figure supplement 1 | AATTTAAGTAATTGGGAAGG | X: 8026448..8026467 | crQY015 |
| *Cbr rex-3* | Figure 10, Figure 10—Figure supplement 1 | GTAGCTAACTCTGTGAAAAT | X: 19468573..19468554 | crQY014 |
| *Cbr rex-3* | Figure 10, Figure 10—Figure supplement 1 | GCGTTGTGGAAGTAGGCAGG | X: 19468752..19468771 | crQY013 |
| *Cel dpy-10* | Figure 5, Figure 8 | GCTACCATAGGCACCACGAG | II: 6711193..6711212 | crispr_bf32 |
| *Cel rex-33* | Figure 12, Figure 13 | TGCCCTACTAAATAAGCGAA | X: 6296617..6296598 | crQY016 |
| *Cel rex-39* | Figure 12 | ACATGTGGAGAACATTATTT | X: 14813548..14813529 | crQY017 |
| *Cel* site 2 | Figure 5 | TTATGTAGTCTCTTTCAGTG | X: 15574657..15574676 | CS568 |
