# Supplementary material for: X-chromosome target specificity diverged between dosage compensation mechanisms of two closely related Caenorhabditis species
Source: eLife. 2023 Mar 23;12:e85413. doi: 10.7554/eLife.85413 (PMC10076027; doi:10.7554/eLife.85413)
Supplement: Supplementary file 5. [file elife-85413-supp5.docx]

| **Target** | **Description** | **Figure** | **Sequence of repair template (5’ to 3’)** | **Related guide** | **Repair ID** |
| --- | --- | --- | --- | --- | --- |
| *Cbr ben-1* | *Cbr* co-injection marker |  | GGGAAGTGATTTCCGACGAGCACGGAATTCAACCTGATGGAACCTACATATGGTGGAGAGAGTGACTTGCAGCTCGAGCGCATCAATGTCTACTACAACG | crispr_bf39 | BF-2036 |
| *Cbr rex-4* | *Cbr rex-4* MEX  (-13.8) scrambled | Figure 9, Figure 9—Figure supplement 1 | GGCCAATTGGCATGGGCTGCCTGCTAACCTTTCCCTGCCTACGCATATTTGATAAGTGATGACTGCGCGGACAAAAGAGGGAAACTAGTGGCCTGCTACCCGAGAAAGAGAGA | crispr_bf77 | BF-2470 |
| *Cbr rex-4* | *Cbr rex-4* MEX II  (-19.09) scrambled | Figure 9, Figure 9—Figure supplement 1 | GCATTCCTCAACCCGCAAAGAGAAGTCAATCGCGCAGATATTGTAATTGTTGCTGCTGCAGTCACTGTGCTCGCTCTGACTGCCTGCTAACCTTTCCCTGCCTACGCATATTTTATATGAACAGGGTGCG | crispr_bf80 | QY070t |
| *Cbr rex-4* | *Cbr rex-4* MEX II  (-19.09) scrambled and MEX (-13.8) scrambled | Figure 9, Figure 9—Figure supplement 1 | GCATTCCTCAACCCGCAAAGAGAAGTCAATCGCGCAGATATTGTAATTGTTGCTGCTGCAGTCACTGTGCTCGCTCTGACTGCCTGCTAACCTTTCCCTGCCTACGCATATTTGATAAGTGATGACTGCG | crispr_bf80 | QY071t |
| *Cbr rex-7* | *Cbr rex-7* MEX  (-18.72) scrambled | Figure 11, Figure 11—Figure supplement 1 | CGTCCTGTTCATTGCAATAAACTGGTGGGAGTTTTTCCAATCTGAGATTTGTGAGCAGTAGTGACAACAGTGATTTAATTTTATGTTCATGAAGTTTTCAGGTTTTTTGCAT | crispr_bf79 | BF-2472 |
| *Cbr rex-7* | *Cbr rex-7* MEX  (-12.26) scrambled, and MEX (-12.58) scrambled | Figure 11, Figure 11—Figure supplement 1 | TCAGGTTTTTTGCATAAAATGCACAATATTCTGAGAAATGTTGTCTTAATCTCGCGTCGTCGTGTTCTCTTCGCACGCAATTTAAGAGTCAGTGTCAGAGGAGAAGACAAATTTGAGGGACCTCTTCTCTTATTTTTTTTTCGCAAAGT | crQY011,  crQY015 | QY087t |
| *Cbr rex-3* | *Cbr rex-3* MEX II  (-20.04) scrambled | Figure 10, Figure 10—Figure supplement 1 | GCAAAGTCAACATGTGTATTTTCCGTGAAAGGACATATGCAAAGGGGTGTCTGTCGAGCTCGCTGTGCTGACGAGAGCAGCTCATAGAGCGTAAATGGGCATTGCCCTCCGCGCAGATACGCGCGTTAAGCCATACCACACATATAACC | crQY013 | QY154t |
| *Cbr rex-3* | *Cbr rex-3* MEX II  (-12.36) scrambled | Figure 10, Figure 10—Figure supplement 1 | TTGATCACACAGTCTTCTTGATGTGCAAGCTAGCTATTTCGAGTAGTTGGAAAATCAAAATTCTATAGTAATACTGTGATAATCATACATCTGTATAGCTACGATGATTTTGAGGAAATGTTTAATAGAAACGTGAAAAAAAGAAATAT | crQY014 | QY155t |

| **Target** | **Description** | **Figure** | **Sequence of repair template (5’ to 3’)** | **Related guide** | **Repair ID** |
| --- | --- | --- | --- | --- | --- |
| *Cel dpy-10* | *Cel* co-injection marker |  | CACTTGAACTTCAATACGGCAAGATGAGAATGACTGGAAACCGTACCGCATGCGGTGCCTATGGTAGCGGAGCTTCACATGGCTTCAGACCAACAGCCTAT | crispr_bf32 | BF-1813 |
| *Cel rex-33* | 3 *Cel* MEX motifs replaced by  *Cbr* MEX motifs in  *Cel rex-33* | Figure 12, Figure 13 | CGTTCAAACAGTCTTTCCTGCAAGCACAGACACTCAAACGTGAGTAATTATTATATGGGCAGGGACACCCAATCGATTGCCCATTTACGAATGCGGCAGGGGGTCACCATAGATAGTAATTGGGAAGGGAAGATTTACCGCCTTTCGCTTATTTAGTAGGGCACGCAAATTAGTATGCTT | crQY016 | QY199t |
| *Cel rex-33* | 3 *Cel* MEX motifs scrambled | Figure 12, Figure 13 | CGTTCAAACAGTCTTTCCTGCAAGCACAGACACTCAAACGTGAGTAATTATTTTAGTCGACGTGACACCCAATCGATTGCCCATTTACATTGCGCTCGAGCGCTCACCATAGATAGGTATGACGCGACGCTGATTTACCGCCTTTCGCTTATTTAGTAGGGCACGCAAATTAGTATGCTT | crQY016 | QY214t |
| *Cel rex-33* | 3 *Cel* MEX motifs with C4G substitution | Figure 13 | CGTTCAAACAGTCTTTCCTGCAAGCACAGACACTCAAACGTGAGTAATTATTTTAAGGGAAGGGACACCCAATCGATTGCCCATTTACATTTGGGGCAGGGGGTCACCATAGATAGGTATCGGGCAGGGAAGATTTACCGCCTTTCGCTTATTTAGTAGGGCACGCAAATTAGTATGCTT | crQY016 | QY245t |
| *Cel rex-33* | 3 *Cel* MEX motifs replaced by  *Cbr* MEX motifs with G7C substitution | Figure 13 | CGTTCAAACAGTCTTTCCTGCAAGCACAGACACTCAAACGTGAGTAATTATTATATGCGCAGGGACACCCAATCGATTGCCCATTTACGAATGCCGCAGGGGGTCACCATAGATAGTAATTGCGAAGGGAAGATTTACCGCCTTTCGCTTATTTAGTAGGGCACGCAAATTAGTATGCTT | crQY016 | QY244t |
| *Cel rex-39* | 2 *Cel* MEX II motifs replaced by  *Cbr* MEX II motifs  in *Cel rex-39* | Figure 12 | TTTCATAACACAACAAGACCGAATAAATATAACACTTATTCCTCCTGCCTACTTCCACAACGCGCGAAGTAACAAGTAACTACTTCGCGCGTTGTGGAAGTAGGCAGGAGGATTTTCAAGAAACATGTGCATGAGTGCATTTCAAAATTC | crQY017 | QY208t |
| *Cel* X site 2 | *Cel* X site 2 insertion of  *Cbr rex-2* | Figure 5 | GAACATGTCGAACACGTACATGACGACTGCTTAAAAGTTGAAAATTTCCCATATCCGTTTCTCATTTTATGTAGTCTCTTTCAGTAATCGTATTCAGCACGTTCGAAAGTACCTTTGTACAAATTTTGAGCTAATTCTCAACGCCCCTCTGAAAACACTTCCCTTGTGAGTTTGAACGGTTTCAGTACAACCATATGGTCAGGGGAACTAAAAAACTAGAAATTCATTACTCGAACATACTGTAGTTATCCCACCATCGCAAATTTGATCGAGTCAACCTCTGCGAAAACGCAAAATAGAAAGGACCACCACACACAAAACGCCCACGTAAACACTGCCCCCTTCCGAGATAAAACATTGAGTGATAACTCTCCTTTTCCGTTTTTCTGAGCGTTTCGCATTTTGGCACGGATCAGTTTCTAATCCACAACTTTAAAAAAATCAAAAATTTTCTTCGAAATTCGAAAGAAAATAAGGAGATTTTTTGACAAGTGAAAAATGAACTCATTCAGTAAGAACGCATATTGTTTCTCAATATTTCTTTTCTATCGTGAAAACGCT TCAACAATCGTTACAAAACTTCATTGGCTACTAACGATTTTGCAATTTTACATTATACTTTGTTTGTGAGTTTCAGGAAACTTGTGAATTCGTTCAAACCCTTCAGAATCAG | CS568 | ER567 |
| *Cel* X site 2 | *Cel* X site 2 insertion of  *Cel rex-32* | Figure 5 | GTCGAACACGTACATGACGACTGCTTAAAAGTTGAAAATTTCCCATATCCGTTTCTCATTTTATGTAGTCTCTTTCAGGAGACAAAAAGGAAAAAATGAAATTGGACAACCCCATAAATTTCATGAATTTTTAAAACTTCTTGCAGGAATATAAATTACTCTAATTAAAGTTTTTTTGTTTGAAAATTTTTTGATAGGCCCAAATACATACTTATCTCTAAAAAAATTACTTTTGAATTCGTTCATTCAATGTGTAAAATCTAAACAAAAGTGACCCCCCTTGTCCAAATATTTTATCCACGAGACTAGGTACACCTCCCTTCGCGATAAATAATTGGTACATCATTTTATCCACAGGGCTACTTCCTCCCTGCGCGATAAATTTAAAATTTTATAACTCTTTGGGTAACTAATTTTGGCAATATTTTCTTATTTTTTCACATCAACAAAAATATTTCAAGTCTAAAAGTTGCAATAAATGCAGTTCAGAGGAAAATTGAGTCGTGTGCGAATAACGAGGAAAAAGACAGACCCATACTTCCTCCCTGCGCGATACGATCTCTATCGACTTTTCTGGTTTTATTGTTTGGCAATGTTTATTAAATTACTCCAAAGATCAAGAAAACAATTATGAAAATTCGAGGAGGTGTAAAATAGAAAATGTTGCAGTTGTGGCTACTAACGATTTTGCAATTTTACATTATACTTTGTTTGTGAGTTTCAGGAAACTTGTGAATTCGTTCAAACCC | CS568 | ER577 |
| *Cel* X  site 2 | *Cel* X site 2 insertion of  *Cbr rex-9* | Figure 5 | GTCGAACACGTACATGACGACTGCTTAAAAGTTGAAAATTTCCCATATCCGTTTCTCATTTTATGTAGTCTCTTTCAGGCAAATTTGAGCTTTCACCTTGATCTCAATTGTACATAATATTTCATGACATTTTTTGTATAAATGTGTTTTCTCATCACTCGATTTTCTTCTGCCAAAAAATAGAGCACTCCATTCCAAAAATAGTATGTCTACGACATTCTCCGCTAATTGTAGTCTTCTGAACACCTCCTTTCGCATGTAAGACGCTGATGGATATAAGATACGAACAGGGTGCAAGGACCCGCGCACGTGCCTTGAATAGACGCTGTTAAAAAGGGCAGACGGCCAGATGGAAGACGTTTCGGAGACAGCGAGGCGGACGAAACGAGTATGTGAGGCCCATTACAACGTCTAATCCATTGGAGGAGAGAGGTTTGCAAAGGGGTGACTGGGGCGACCAGCATTTTTATGTTTGATGGATGTGACCGGGAAAATGACGGGTTGTCATCAGTGCAAGGTGACACAAAAAAACCGACAGTAGAATGGTAGTTTTTTTTTGCAATTTAAACGTTATCCATCATATTACGGTAGTGGAGGAGTAGTGACACCGCTAAATGCATCTGATAAGTTTTATCAGGGTAGTCAAGATGATTTTTGCAACAAATTTTGAACTGTACTTTGTGGCTACTAACGATTTTGCAATTTTACATTATACTTTGTTTGTGAGTTTCAGGAAACTTGTGAATTCG | CS568 | ER581 |
| *Cel X  site 2* | *Cel* X site 2 insertion of  *Cbr rex-7* | Figure 5 | GTCGAACACGTACATGACGACTGCTTAAAAGTTGAAAATTTCCCATATCCGTTTCTCATTTTATGTAGTCTCTTTCAGGTCAGTTGATCACTTTCTGATAATTCTTACATGAACCAAAACAAATTTGCTTGAAATTGAAAGTTGAACTGCTCATTTCATTAGAGTGTTTACGCTATTTTTCGAAAATTTGTTTAAAAGTATACT | CS568 | ER586 |
|  |  |  | TGAAACGTAGCAAAAAGCTTACTGAAAGAAAACGGAAATGTTTCATTTTAATTGATTTTGTCCGGTGTTTTTGTTAAATCAGCTTTATCGTTTCCTGAAATTTTCGAGATTTGCCCGACCGTCCTGTTCATTGCAATAAACTGGTGGGAGTTTTTCCAATCTGAGATTTTATATGGGCAGGGACAACAGTGATTTAATTTTATGTTCATGAAGTTTTCAGGTTTTTTGCATAAAATGCACAATATTCTGAGAAATGTTGTCTTAATCCCCTGCCGCATTCTTCTCTTCGCACGCAATTTAAGTAATTGGGAAGGGGGAGAAGACAAATTTGAGGGACCTCTTCTCTTATTTTTTTTTCGCAAAGTAGCAGCAATGAATTTTGCGATAAAAAGGATTCTACAAGTCGTTTTCTATTTTCCTTCCTTTTCCAGTGGAAACTCTTCAAACAGACCAAATATTTTGCACTCTGTTGTCATGTGGCTACTAACGATTTTGCAATTTTACATTATACTTTGTTTGTGAGTTTCAGGAAACTTGTGAATTCGTTCAAACCC |  |  |
| *Cel X  site 2* | *Cel* X site 2 insertion of  *Cbr rex-4* | Figure 5 | GTCGAACACGTACATGACGACTGCTTAAAAGTTGAAAATTTCCCATATCCGTTTCTCATTTTATGTAGTCTCTTTCAGGTATGAAATTTGAAGTGTTTGAATATATACTCTCTGCACTCCGACTATTTTACAGTGCGACTGGCAAATCTCACTGAATGAGTAAGGTCAACTGACAATCAACAAAGATATAATTCCAAAGTTTTCTCATTTCTTGAATATAAGTATTTGATGTTTGAAAAAATCGGCATTTCTTGCAAATGTACTGAATGAATTGTAATCTTGGTCAGACATCGTATGGCATTCCTCAACCCGCAAAGAGAAGTCAATCGCGCAGATATTGTAATTGTTCTCTGCGCGTATGGCCAATTGGCATGGGCTGCCTGCTAACCTTTCCCTGCCTACGCATATTTTATATGAACAGGGTGCGCGGACAAAAGAGGGAAACTAGTGGCCTGCTACCCGAGAAAGAGAGAAATGCAACGTTTAATAAACCGATGACGAGCAGGCAACGTGCCCCTTCCGATTTGAATGGCGTTTCAAAATCAGAGTCAGACTTTCTGCGAAGATAGTTTTTGTAAGCGCTTCGAGGGCAATGGAATCCTAAAATGATCAATTTTAATGAAATCGGGATGTAATGTAGGTAGAAATCTAGATCTACGTAGAACCAGGAACTATGTTCTGTGGCTACTAACGATTTTGCAATTTTACATTATACTTTGTTTGTGAGTTTCAGGAAACTTGTGAATTCG | CS568 | ER587 |
| *Cel X  site 2* | *Cel* X site 2 insertion of  *Cbr rex-5* | Figure 5 | GTCGAACACGTACATGACGACTGCTTAAAAGTTGAAAATTTCCCATATCCGTTTCTCATTTTATGTAGTCTCTTTCAGGTAGTTTTTCGAAAGAACCGCCCAAATTTTGAACTAGACTTGAAAATATTTTCGCGCGTTTAAAAACTTCATTTACTCAGAGTTACATCTTTCAACTTTACGAAGAAAGCATATGAAAGCGTTTTCACGTCTCGTTTCTCTAAATCTCCATTTCCATTTTTGATCCTTTCTCGTGCGGATTTTCTCAACGCCCAAAGAAGTGAAAAATATTTCTTTGAAAAAGAAAAATAGACATTGACGGAGGACAATTATTTTAGAGAAAAACCAACTAACTCTACGAAAAGGTTATATAGGCAAGCATATCAAAAATCAGATTTACATCAAAATTGCAGAACAAAAGCAGAGAAAATCTGGTTCAACGGGATGCCAAGAATTTTCTCTATGTGGGTTTTCAGTTTCTTAAAAGGGATCAACTTCAAATATTATAGAAACCATTTGAGCTTTTAACTTTTCCATAATTGGTCATTGAAAGTTTGCTTGATTATTAAAAATCAAAAACCAAATGACTTTCTTGTACGGCTTTCATTCCGTCAG AACCCTAATGAAAATATAGAATTTATAAAAGTACATTTATTAGGTTGGAAGAAAAGTAACTGTCCACTGTGGCTACTAACGATTTTGCAATTTTACATTATACTTTGTTTGTGAGTTTCAGGAAACTTGTGAATTCG | CS568 | ER588 |
