# Supplementary material for: X-chromosome target specificity diverged between dosage compensation mechanisms of two closely related Caenorhabditis species
Source: eLife. 2023 Mar 23;12:e85413. doi: 10.7554/eLife.85413 (PMC10076027; doi:10.7554/eLife.85413)
Supplement: Supplementary file 6. [file elife-85413-supp6.docx]

| **Figure** | **ID** | **Description** | **Forward Primer** | **Forward Primer**  **Sequence** | **Reverse Primer** | **Reverse Primer**  **Sequence** | **Probe Sequence** |
| --- | --- | --- | --- | --- | --- | --- | --- |
| Figure 12C | pKB460 | WT *rex-39* (reverse complement) | kb416 | CGATACATTTGTTTTTATTAAATATCTACATTTTCTCG | kb417r | TTTCTGAAAAAATTGAAAGAATCTTGCTTAAAAATG | TTTCTGAAAAAATTGAAAGAATCTTGCTTAAAAATGAAAATGTTAACCTTAGCTTAAACTGGCAAGACCTCAATTCCTGTTCAGCATGAAAAACTCTTCGCATTATGAACTTTCGTTTCCGAATGTTATCCTGGTATGCTTCCACTCGAGTGTTACACTTTTCCATGTTCTATTATAATACAATTGAATTTTATTACTTCATGTCAAAAAACTCACAACTAAATCAAACTCTTCAACCAGCTGTCTCAATTTCTCAACTCGCCGTTTTTGAAAGAAATCCTGCAATAAATCCGTAAAATTTTGCCTAATAAGTCGAATTTCAGCAGCAAGGTTGTCGTCAAATGATGCTTTGCAGGCTGAAAATAAGCTTTTAGAAATAGTGGCAGGTTCATTACTTTTTTTTCATAACACAACAAGACCGAATAAATATAACACTTATTCAGCTTGCACCAATTACGTTAGCCACGAGAAACAAGTAACTACATGTGGAGAACATTATTTGGGCACGTGTATTTTCAAGAAACATGTGCATGAGTGCATTTCAAAATTCAAATGTGGATTTTCGAGAAAATGTAGATATTTAATAAAAACAAATGTATCG |
| Figure 12C | pKB468 | *rex-39;*  both MEX II mutated | kb416 | CGATACATTTGTTTTTATTAAATATCTACATTTTCTCG | kb417r | TTTCTGAAAAAATTGAAAGAATCTTGCTTAAAAATG | TTTCTGAAAAAATTGAAAGAATCTTGCTTAAAAATGAAAATGTTAACCTTAGCTTAAACTGGCAAGACCTCAATTCCTGTTCAGCATGAAAAACTCTTCGCATTATGAACTTTCGTTTCCGAATGTTATCCTGGTATGCTTCCACTCGAGTGTTACACTTTTCCATGTTCTATTATAATACAATTGAATTTTATTACTTCATGTCAAAAAACTCACAACTAAATCAAACTCTTCAACCAGCTGTCTCAATTTCTCAACTCGCCGTTTTTGAAAGAAATCCTGCAATAAATCCGTAAAATTTTGCCTAATAAGTCGAATTTCAGCAGCAAGGTTGTCGTCAAATGATGCTTTGCAGGCTGAAAATAAGCTTTTAGAAATAGTGGCAGGTTCATTACTTTTTTTTCATAACACAACAAGACCGAATAAATATAACACTTATTCTTTCATTGTTTGCGCGAGATATGCAAAGAAACAAGTAACTACAAAAAAGATGAACGCGCTCCTTTTTTCTATTTTCAAGAAACATGTGCATGAGTGCATTTCAAAATTCAAATGTGGATTTTCGAGAAAATGTAGATATTTAATAAAAACAAATGTATCG |

| **Figure** | **ID** | **Description** | **Forward Primer** | **Forward Primer**  **Sequence** | **Reverse Primer** | **Reverse Primer**  **Sequence** | **Probe Sequence** |
| --- | --- | --- | --- | --- | --- | --- | --- |
| Figure 12C | pKB1015 | *rex-39;*  both MEX II mutated to *Cbr* MEX II | kb416 | CGATACATTTGTTTTTATTAAATATCTACATTTTCTCG | kb417r | TTTCTGAAAAAATTGAAAGAATCTTGCTTAAAAATG | TTTCTGAAAAAATTGAAAGAATCTTGCTTAAAAATGAAAATGTTAACCTTAGCTTAAACTGGCAAGACCTCAATTCCTGTTCAGCATGAAAAACTCTTCGCATTATGAACTTTCGTTTCCGAATGTTATCCTGGTATGCTTCCACTCGAGTGTTACACTTTTCCATGTTCTATTATAATACAATTGAATTTTATTACTTCATGTCAAAAAACTCACAACTAAATCAAACTCTTCAACCAGCTGTCTCAATTTCTCAACTCGCCGTTTTTGAAAGAAATCCTGCAATAAATCCGTAAAATTTTGCCTAATAAGTCGAATTTCAGCAGCAAGGTTGTCGTCAAATGATGCTTTGCAGGCTGAAAATAAGCTTTTAGAAATAGTGGCAGGTTCATTACTTTTTTTTCATAACACAACAAGACCGAATAAATATAACACTTATTCCTCCTGCCTACTTCCACAACGCGCGAAGTAACAAGTAACTACTTCGCGCGTTGTGGAAGTAGGCAGGAGGATTTTCAAGAAACATGTGCATGAGTGCATTTCAAAATTCAAATGTGGATTTTCGAGAAAATGTAGATATTTAATAAAAACAAATGTATCG |
| Figure 12F, Figure 13C | pKB1023 | WT *rex-33* (reverse complement) | kb221 | AGATTTTGCGGTCAAATTGAGG | kb222r | GTCAGAAAGGGTATACGTTCCAAG | CTTGGAACGTATACCCTTTCTGACAAAAAACTATTTATAACGTCATGTTTTGATCATAACGTTTCATAACGTTCAAAATTATTGTTAGCTCAATTTATTTTGGACAACTCTTGCAAATTCTTAAATTTATAAACCATGCGATATTTGCAAACTCCAAATGTGTCCAAAAAAGGGCCCGTGGTTAATTTATTCGTGTTTTATCGTGTTTCTAACAGTTAAGCATACTAATTTGCGTGCCCTACTAAATAAGCGAAAGGCGGTAAATCTTCCCTGCGCGATACCTATCTATGGTGACCCCCTGCGCCAAATGTAAATGGGCAATCGATTGGGTGTCCCTTCGCTTAAAATAATTACTCACGTTTGAGTGTCTGTGCTTGCAGGAAAGACTGTTTGAACGGACAGGGCAGGCTTTGGCAGCAACACACATTGAGTATCGGGCAGAAAGTCGTTTTCGGAAAACTGAAAAAAAAATCTTAAACTATACAAGTGGAGTAGAGACATTTTGCAGTAACTTTTGAAATATATAAAATTACATGTATTGTTTCCATATAACATAGATAAAAGTTACTTTAAATTCAACCTCAATTTGACCGCAAAATCT |
| Figure 12F, Figure 13C | pKB1022 | *rex-33* with all 3 MEX motifs scrambled | kb221 | AGATTTTGCGGTCAAATTGAGG | kb222r | GTCAGAAAGGGTATACGTTCCAAG | CTTGGAACGTATACCCTTTCTGACAAAAAACTATTTATAACGTCATGTTTTGATCATAACGTTTCATAACGTTCAAAATTATTGTTAGCTCAATTTATTTTGGACAACTCTTGCAAATTCTTAAATTTATAAACCATGCGATATTTGCAAACTCCAAATGTGTCCAAAAAAGGGCCCGTGGTTAATTTATTCGTGTTTTATCGTGTTTCTAACAGTTAAGCATACTAATTTGCG |
|  |  |  |  |  |  |  | TGCCCTACTAAATAAGCGAAAGGCGGTAAATCAGCGTCGCGTCATACCTATCTATGGTGAGCGCTCGAGCGCAATGTAAATGGGCAATCGATTGGGTGTCACGTCGACTAAAATAATTACTCACGTTTGAGTGTCTGTGCTTGCAGGAAAGACTGTTTGAACGGACAGGGCAGGCTTTGGCAGCAACACACATTGAGTATCGGGCAGAAAGTCGTTTTCGGAAAACTGAAAAAAAAATCTTAAACTATACAAGTGGAGTAGAGACATTTTGCAGTAACTTTTGAAATATATAAAATTACATGTATTGTTTCCATATAACATAGATAAAAGTTACTTTAAATTCAACCTCAATTTGACCGCAAAATCT |
| Figure 12F, Figure 13C | pKB1021 | *rex-33* with all 3 MEX motifs mutated to *Cbr* MEX motifs | kb221 | AGATTTTGCGGTCAAATTGAGG | kb222r | GTCAGAAAGGGTATACGTTCCAAG | CTTGGAACGTATACCCTTTCTGACAAAAAACTATTTATAACGTCATGTTTTGATCATAACGTTTCATAACGTTCAAAATTATTGTTAGCTCAATTTATTTTGGACAACTCTTGCAAATTCTTAAATTTATAAACCATGCGATATTTGCAAACTCCAAATGTGTCCAAAAAAGGGCCCGTGGTTAATTTATTCGTGTTTTATCGTGTTTCTAACAGTTAAGCATACTAATTTGCGTGCCCTACTAAATAAGCGAAAGGCGGTAAATCTTCCCTTCCCAATTACTATCTATGGTGACCCCCTGCCGCATTCGTAAATGGGCAATCGATTGGGTGTCCCTGCCCATATAATAATTACTCACGTTTGAGTGTCTGTGCTTGCAGGAAAGACTGTTTGAACGGACAGGGCAGGCTTTGGCAGCAACACACATTGAGTATCGGGCAGAAAGTCGTTTTCGGAAAACTGAAAAAAAAATCTTAAACTATACAAGTGGAGTAGAGACATTTTGCAGTAACTTTTGAAATATATAAAATTACATGTATTGTTTCCATATAACATAGATAAAAGTTACTTTAAATTCAACCTCAATTTGACCGCAAAATCT |
| Figure 13C | pKB1026 | *rex-33* with all 3 MEX motifs mutated to *Cbr* MEX motifs with G7C change | kb221 | AGATTTTGCGGTCAAATTGAGG | kb222r | GTCAGAAAGGGTATACGTTCCAAG | CTTGGAACGTATACCCTTTCTGACAAAAAACTATTTATAACGTCATGTTTTGATCATAACGTTTCATAACGTTCAAAATTATTGTTAGCTCAATTTATTTTGGACAACTCTTGCAAATTCTTAAATTTATAAACCATGCGATATTTGCAAACTCCAAATGTGTCCAAAAAAGGGCCCGTGGTTAATTTATTCGTGTTTTATCGTGTTTCTAACAGTTAAGCATACTAATTTGCGTGCCCTACTAAATAAGCGAAAGGCGGTAAATCTTCCCTTCGCAATTACTATCTATGGTGACCCCCTGCGGCATTCGTAAATGGGCAATCGATTGGGTGTCCCTGCGCATATAATAATTACTCACGTTTGAGTGTCTGTGCTTGCAGGAAAGACTGTTTGAACGGACAGGGCAGGCTTTGGCAGCAACACACATTGAGTATCGGGCAGAAAGTCGTTTTCGGAAAACTGAAAAAAAAATCTTAAACTATACAAGTGGAGTAGAGACATTTTGCA |
|  |  |  |  |  |  |  | GTAACTTTTGAAATATATAAAATTACATGTATTGTTTCCATATAACATAGATAAAAGTTACTTTAAATTCAACCTCAATTTGACCGCAAAATCT |
| Figure 13C | pKB1028 | *rex-33* with C4G mutated in all 3 MEX motifs | kb221 | AGATTTTGCGGTCAAATTGAGG | kb222r | GTCAGAAAGGGTATACGTTCCAAG | CTTGGAACGTATACCCTTTCTGACAAAAAACTATTTATAACGTCATGTTTTGATCATAACGTTTCATAACGTTCAAAATTATTGTTAGCTCAATTTATTTTGGACAACTCTTGCAAATTCTTAAATTTATAAACCATGCGATATTTGCAAACTCCAAATGTGTCCAAAAAAGGGCCCGTGGTTAATTTATTCGTGTTTTATCGTGTTTCTAACAGTTAAGCATACTAATTTGCGTGCCCTACTAAATAAGCGAAAGGCGGTAAATCTTCCCTGCCCGATACCTATCTATGGTGACCCCCTGCCCCAAATGTAAATGGGCAATCGATTGGGTGTCCCTTCCCTTAAAATAATTACTCACGTTTGAGTGTCTGTGCTTGCAGGAAAGACTGTTTGAACGGACAGGGCAGGCTTTGGCAGCAACACACATTGAGTATCGGGCAGAAAGTCGTTTTCGGAAAACTGAAAAAAAAATCTTAAACTATACAAGTGGAGTAGAGACATTTTGCAGTAACTTTTGAAATATATAAAATTACATGTATTGTTTCCATATAACATAGATAAAAGTTACTTTAAATTCAACCTCAATTTGACCGCAAAATCT |
| Figure 12C, Figure 12F | pKB212 | np 1 | kb204 | ATTTGTATCAAATCAAAGAGCAGG | kb183r | GCGGTAACTGCTAGTTTTCAGG | ATTTGTATCAAATCAAAGAGCAGGACACGGTTGTTGCTTCAATCTACTATGTGCTAACGTTTATTTTCGAAATGACAGCATTCTTTGTTATTAACAAAATGAATATCCCTTTCTTTTCGGTAATTTTATGGTAGTTTACCGAGTAGAGACATTCAAATTTTAGGAGGACAATTTTCTGGATCACGTGGGAATGGATAATGATAACCAGGTAATGGTCACATTGTGTTGATGTAAAAGAACAAGAAAATACAAAAAAGAAAGCAAGATCTTTTAATCAAAGTCCAAGATTTGTTTCTTCATGAAATCTGTGGAAGTTGTTGGTTAAAGTACAACCCAGACCACGAGGGACTTGAGTTATCTCGTCATTTAATTTTGTTTGATTTTCCGGTAGTTATGTATGTAAACATCAGAATATTCCATTTGTCTGTAGCTCATAATGATGCTGATAATAAATTTGTTATGCACTAATGACGAAAGCTAATGATTATTTTATCGTCTATTATTTTTCGCATCTTTCAACTTCCTGGTATCTTGTTTTCTAAAATTATATTTTCATATTTTCTCGTTGCTGCCAAAAGTCCTGAAAACTAGCAGTTACCGC |
